# Aberrant myocardial sheetlet mobility in hypertrophic cardiomyopathy detected using in vivo cardiovascular magnetic resonance diffusion tensor imaging

**DOI:** 10.1186/1532-429X-16-S1-P338

**Published:** 2014-01-16

**Authors:** Pedro Ferreira, Philip J Kilner, Laura-Ann McGill, Sonia Nielles-Vallespin, Andrew D Scott, Bruce S Spottiswoode, Xiaodong Zhong, Siew Y Ho, Karen McCarthy, Tevfik Ismail, Peter Gatehouse, Ranil Silva, Alexander Lyon, Sanjay K Prasad, David Firmin, Dudley J Pennell

**Affiliations:** 1 Cardiovascular Biomedical Research Unit, Royal Brompton Hospital, London, UK; 2 Cardiovascular Magnetic Resonance Unit, Royal Brompton Hospital, London, UK; 3 National Institutes of Health, Bethesda, Maryland, USA; 4 National Heart and Lung Institute, London, UK; 5 Siemens Healthcare, Chicago, Illinois, USA; 6 Siemens Healthcare, Atlanta, Georgia, USA

## Background

Background: Cardiac diffusion tensor imaging (cDTI) contains information on cross-myocyte components of intramyocardial water diffusion. Assuming these to be constrained by the sheetlet and shear layer microstructure of left ventricular myocardium [1], we hypothesized that cDTI at two cardiac phases would identify changing sheetlet orientations and abnormalities in hypertrophic cardiomyopathy (HCM).

## Methods

We performed cDTI in vivo at 3 Tesla at end-systole and late-diastole in 11 healthy controls and 11 patients with HCM, with previous late gadolinium enhancement (LGE) for detection of fibrosis.

## Results

Voxel-wise analysis of diffusion tensors relative to ventricular coordinates showed transmural changes of helix-angle, with no differences between phases or between HCM and controls. In controls, the orientation of the second eigenvector of diffusion (E2A), changed from more wall-parallel in diastole to more wall-perpendicular in systole, in accord with the predicted reorientations of sheetlet populations. HCM hearts showed markedly abnormal global E2A in diastole consistent with impaired relaxation (46.8° vs 24.0° controls, p < 0.001), and minor abnormal global E2A in systole consistent with hypercontraction (63.9° vs 56.4° controls, p = 0.026). In hypertrophic regions, sheetlets retained relatively systolic orientations in diastole, independent of fibrosis, which differed from regions of normal wall thickness (LGE present 57.8°, p = 0.0028, LGE absent 54.8°, p = 0.0022 vs normal thickness 38.1°).

## Conclusions

In vivo DTI quantifies cross-myocyte diffusion. We are potentially showing impaired in vivo diastolic reorientation of sheetlet populations in HCM, although further investigation is required as myocardial strain is a possible confounder. Current work includes the measurement of 3D strain in all subjects for assessment of contractility and for strain correction of the diffusion tensor [2]. The persistence of a systolic conformation may provide novel phenotypic insight into diastolic abnormalities arising from sarcomeric dysfunction, with potential therapeutic implications.

## Funding

This work was supported by the National Institute of Health Research Cardiovascular Biomedical Research Unit at the Royal Brompton Hospital and Imperial College, London.

**Figure 1:**
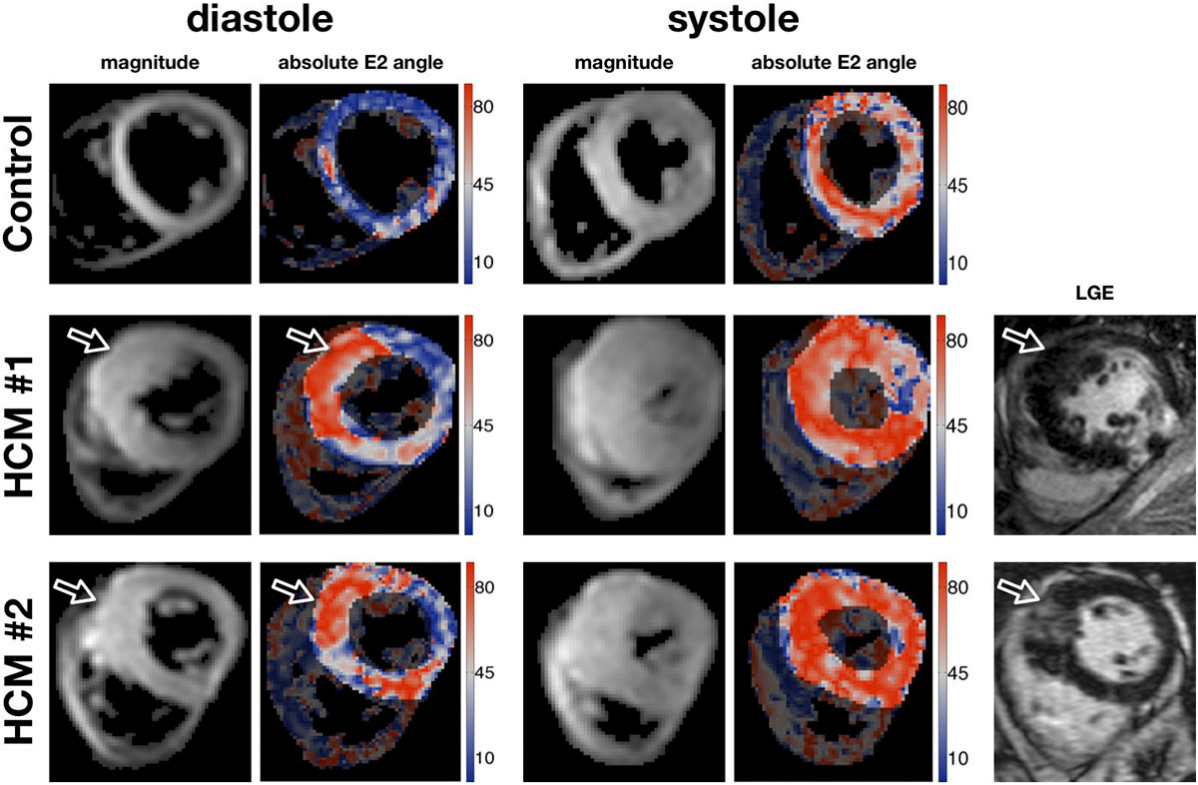
**Averaged magnitude image and the respective E2 angle maps for a control and 2 HCM examples with anteroseptal hypertrophy at the 2 imaged cardiac phases**. Additionally the 2 HCM examples also have on the right the matching LGE images. E2A differences between controls and HCM can be seen in the hypertrophied regions, mainly in the diastolic phase. The non-hypertrophic lateral wall in both HCM hearts approaches the absolute E2A angles measured in the control heart.

**Figure 2:**
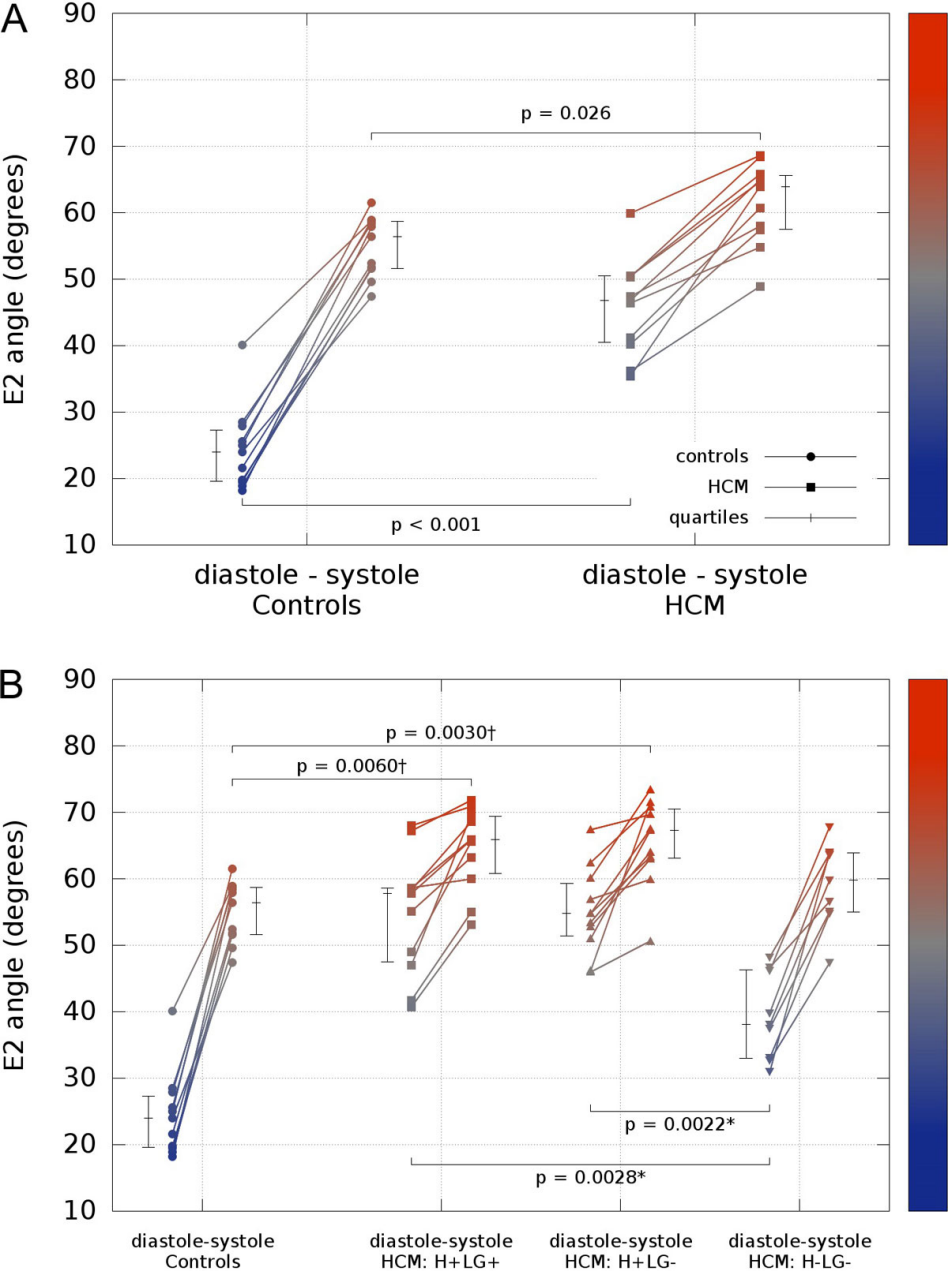
**Scatter plots showing the E2 mobility (mean absolute E2 angle change between diastole and systole) for all subjects at the two imaged cardiac phases**. A) Global mean E2A values. B) Global controls vs HCM cohort with the myocardium divided into three different regions: regions with hypertrophy and LGE (H+LG+), regions with hypertrophy but no LGE (H+LG-), and regions with no hypertrophy or LGE (H-LG-). In all plots the median and interquartile range are shown. Of note, it shows the most abnormal orientations, inclined steeply inward from the wall plane with low mobility, in the hypertrophic regions, whether not there is LGE evidence of fibrosis. *P-value multiplied by 2 for Bonferroni correction for 2 tests. †P-value multiplied by 3 for Bonferroni correction for 3 tests.

## References

[B1] Hales Prog Biophys Mol Biol 2012 110 319 10.1016/j.pbiomolbio.2012.07.014 23043978 PMC3526796

[B2] Reese JMRB 1996 112 253 10.1006/jmrb.1996.0139

